# Germaphobia! Does Our Relationship With and Knowledge of Biodiversity Affect Our Attitudes Toward Microbes?

**DOI:** 10.3389/fpsyg.2021.678752

**Published:** 2021-06-30

**Authors:** Jake M. Robinson, Ross Cameron, Anna Jorgensen

**Affiliations:** ^1^Department of Landscape Architecture, The University of Sheffield, Sheffield, United Kingdom; ^2^inVIVO Planetary Health, Worldwide Universities Network, Jersey City, NJ, United States; ^3^The Healthy Urban Microbiome Initiative (HUMI), Adelaide, SA, Australia

**Keywords:** microbiome, microorganisms, COVID-19, germaphobia, mysophobia, nature connectedness, nature relatedness

## Abstract

Germaphobia – a pathological aversion to microorganisms – could be contributing to an explosion in human immune-related disorders *via* mass sterilization of surfaces and reduced exposure to biodiversity. Loss of biodiversity and people’s weaker connection to nature, along with poor microbial literacy may be augmenting the negative consequences of germaphobia on ecosystem health. In this study, we created an online questionnaire to acquire data on attitudes toward, and knowledge of microbes. We collected data on nature connectedness and interactions with nature and explored the relationships between these variables. Although the study had an international reach (*n* = 1,184), the majority of responses came from England, United Kingdom (*n* = 993). We found a significant association between attitudes toward microbes and both duration and frequency of visits to natural environments. A higher frequency of visits to nature per week, and a longer duration spent in nature per visit, was significantly associated with positive attitudes toward microbes. We found no association between nature connectedness and attitudes toward microbes. We found a significant relationship between knowledge of “lesser known” microbial groups (e.g., identifying that fungi, algae, protozoa, and archaea are microbes) and positive attitudes toward microbes. However, we also found that people who identified viruses as being microbes expressed less positive views of microbes overall–this could potentially be attributed to a “COVID-19 effect.” Our results suggest that basic microbial literacy and nature engagement may be important in reducing/preventing germaphobia-associated attitudes. The results also suggest that a virus-centric phenomenon (e.g., COVID-19) could increase broader germaphobia-associated attitudes. As the rise of immune-related disorders and mental health conditions have been linked to germaphobia, reduced biodiversity, and non-targeted sterilization, our findings point to a feasible strategy to potentially help ameliorate these negative consequences. Further research is needed, but greater emphasis on microbial literacy and promoting time spent in nature could potentially be useful in promoting resilience in human health and more positive/constructive attitudes toward the foundations of our ecosystems—the microorganisms.

## Introduction

Germaphobia—also known as “mysophobia”—is the pathological fear of, and aversion to dirt and microorganisms (henceforth referred to as “microbes”) (Zemke et al., 2015). The rise of germaphobia has likely been influenced by decades of advertising campaigns creating negative perceptions of microbes, and falsely prompting mass (non-targeted) sterilization of surfaces to achieve “safe” human environments (Timmis et al., 2019). Symptoms of germaphobia include avoiding certain “dirty” environments (e.g., soil) due to perceived to fear of microbial exposure, excessively washing hands, over-use of sanitizers and antibiotics (Qadir and Yameen, 2019). However, far less than 1% of the microbes on the planet are human pathogens (Zobell and Rittenberg, 2011; Balloux and van Dorp, 2017). Moreover, germaphobia may have contributed to the current explosion in human immune-related disorders (such as diabetes, asthma, and inflammatory bowel disease) (Jun et al., 2018; Timmis et al., 2019). This is thought to be attributed to the notion that exposure to environmental microbiomes—the diverse network of microbes in a given environment—plays an important role in human health (Rook et al., 2003; Dannemiller et al., 2014; Stein et al., 2016; Arleevskaya et al., 2019; Liddicoat et al., 2019; Selway et al., 2020). Indeed, from a young age, exposure to a diverse range of environmental microbes is considered to be essential for the assembly of our microbiome and the training and regulation of our immune systems (Flies et al., 2020; Renz and Skevaki, 2020; Roslund et al., 2020). A stable and functional human microbiome is colonized following birth. Firstly by the mother’s skin and breast milk, and later supplemented from visitors, pets, biodiverse environments, and a “normal dirty” (not overly cleaned) home environment (DeWeerdt, 2018). Germaphobia and associated overly-clean disposition (whilst recognizing targeted hygiene is essential) could conceivably inhibit all of these activities (e.g., avoiding playing in soil or staying away from animals), and if the microbiome assembly process is derailed, the negative health consequences such as immune dysfunction, could be long-term (Gensollen et al., 2016; Renz and Skevaki, 2020). In relation to the current COVID-19 pandemic – a situation that could conceivably increase germaphobia – in addition to being hygienic, we need to promote the concept that the majority of microbes are in fact innocuous and/or beneficial to human health *via* immunoregulation and other functional roles (Rook, 2013). Indeed, through the modulation of host immune responses, the gut microbiome may even have a direct role in regulating COVID-19 severity (Yeoh et al., 2021).

Microbial communities and their interactions also play essential roles in carbon and nutrient cycling, climate regulation, animal and plant health, and global food security (Cavicchioli et al., 2019; Li et al., 2020; Trivedi et al., 2020). Therefore, microbial biodiversity is of vital importance for the ability of ecosystems to simultaneously provide multiple ecosystem services (Guerra et al., 2020). Consequently, ongoing degradation of microbial communities likely poses an important threat to global macro-level biodiversity and to human societies across the planet (Cavicchioli et al., 2019). Loss of biodiversity and our affective, cognitive and experiential connection with the natural world (also known as “nature connectedness”), along with poor microbial literacy (such as awareness of the different types of microbes and their importance) and germaphobia, may be detrimental to ecosystem health (Cavicchioli et al., 2019; Robinson and Breed, 2020). Studies have suggested that environmental knowledge (particularly of macro-ecological features) can play a role in fostering pro-ecological attitudes and behaviors (Sat Gungor et al., 2018; Choe J. H. et al., 2020), while other suggest knowledge is not an important factor (Qomariah and Prabawani, 2020). A recent study investigated the factors that account for pro-ecological behaviors, and found that nature connectedness, nature experiences (time spent in nature and nature engagement) and nature-based knowledge and attitudes explained 70% of the variation in people’s actions for nature (Richardson et al., 2020). Other studies have shown that connectedness to nature and frequency of visits to nature is linked to pro-ecological behaviors (Collado et al., 2015; Duron-Ramos et al., 2020). Recent work suggested that outdoor nature experiences can help overcome fears of “creepy crawlies” such as insects and snakes and can help develop respectful and positive attitudes toward nature (Hosaka et al., 2017; Chawla, 2020).

Is our diminishing connection with (the rest of) the natural world helping to drive germaphobia-associated attitudes (which may subsequently affect behaviors)? To our knowledge, no studies have investigated the relationship between nature engagement (duration and frequency in nature), nature connectedness and attitudes toward the invisible constituents of nature (i.e., microorganisms). Furthermore, no studies have explored whether there is a relationship between basic knowledge of microorganisms and attitudes toward microorganisms.

In this study, we used an online questionnaire to acquire data on attitudes toward microbes. We collected data on respondents’ nature engagement (including typical duration and frequency of visits to nature), and data on nature connectedness using the Nature Relatedness 6 Scale—a validated psychological instrument (Nisbet and Zelenski, 2013). To gauge respondents’ basic knowledge of microbes, we asked them to select all of the organisms (from a list) that they considered to be microbes. The relationships between these variables (i.e., between nature connectedness, nature engagement and attitudes toward microbes; and between basic microbial literacy and attitudes toward microbes) were then assessed using a range of statistical methods including logistic regression models, Mann–Whitney *U* tests, and 2-sample tests for equality of proportions with continuity correction in R.

The primary objectives of this study were to: (a) assess whether people’s patterns of exposure to nature associated with their attitudes toward microbes (i.e., a positive or negative view); (b) assess whether people’s level of subjective connectedness to nature associated with their attitudes toward microbes; and, (c) investigate whether basic knowledge of microbial groups (e.g., identifying that fungi, algae, protozoa, and archaea are also microbes) associated with attitudes toward microbes.

Gaining a better understanding of the factors that may aid in reducing/preventing germaphobia-associated attitudes (e.g., negative attitudes that may influence subsequent behaviors) could help to inform environmental and public health policy. For example, improving microbial literacy and promoting campaigns that seek to reconnect humans with the wider biotic community could potentially bring value to both human and environmental health. Microbes are the foundations of our ecosystems and are essential to the survival of all life on Earth (Cavicchioli et al., 2019). Targeted hygiene approaches and continued efforts to control infectious diseases are undoubtedly vital. However, germaphobia (and associated actions such as soil/nature avoidance, and mass sterilization of the environment) only serves to inhibit a more nuanced awareness of, and mutually-advantageous relationship with these diverse, underappreciated, and indispensable lifeforms.

## Materials and Methods

### Online Questionnaire

We produced a research questionnaire using the Smart Survey online software (Smart Survey, 2020). The questionnaire included 21 multi-format questions (Supplementary Appendix A). The questions were devised to gather data on respondents based on four variables: (1) nature engagement (*via* determining frequency and duration in nature); (2) nature connectedness; (3) attitudes toward microbes; and, (4) basic knowledge of microbes. The online survey was active between April and July 2020.

#### Nature Engagement

As the study was conducted during the COVID-19 pandemic, we asked participants to provide answers by referring to their typical patterns of visiting nature before the pandemic. For example, the following questions were asked: “how many times would you visit any natural environments (e.g., parks, woodlands, and the beach) in a typical week before the COVID-19 pandemic?”; and “Approximately how long would you spend in any natural environment per visit before the COVID-19 pandemic?” For this study “natural environments” and/or “nature” were considered to be less anthropogenic/built-up environments, typically containing a large proportion of vegetation and wildlife such as woodlands, parks, and meadows.

#### Nature Connectedness

We asked participants to answer questions regarding how emotionally and cognitively connected they felt to nature using the Nature Relatedness Scale (NR-6) (Nisbet and Zelenski, 2013; Kettner et al., 2019). The NR-6 comprises six questions, and answers are recorded using a 1–5 Likert scale. Examples of questions include “My relationship to nature is an important part of who I am,” “My ideal vacation spot would be a remote, wilderness area,” and “I feel very connected to all living things and the earth.” Items were averaged, and higher scores indicated stronger subjective connectedness to nature. This validated instrument has been used in several previous environmental psychology studies (Nisbet and Zelenski, 2013; Obery and Bangert, 2017; Whitburn et al., 2020). We also asked several pilot-tested questions regarding typical exposure to nature such as duration and frequency of visits to natural environments.

#### Attitudes Toward Microbes

To acquire data on respondents’ attitudes toward microbes, we devised a pilot-tested word-association measure using three categories: positive association, neutral association, and negative association. To reduce potential bias, the categories were not revealed to the respondents and each category contained five randomly-ordered words, displayed as one amalgamated list (Supplementary Appendix A). In the positive category, respondents could choose from words such as “essential” and/or “beneficial.” In the neutral category respondents could choose from words such as “nature” and/or “mobile.” In the negative category respondents could choose from words such as “disease” and/or “nuisance.” Respondents were asked to select a total of three words that best reflected their view of microbes. We also used the questions “do you consider microbes to be good?; bad?; some are good, some are bad?; or, neither are good or bad?,” the resulting positive and negative categories were used in the models to explore the influence of nature connectedness. To gauge respondents’ basic knowledge of microbes, we asked them to select all of the organisms that they considered to be microbes. The list included bacteria, viruses, fungi, algae, protozoa, and archaea. Due to the current COVID-19 pandemic, which is of viral origin, we separated out viruses in some of the analyses in case they affected people’s overall perception of microbes.

#### Demographic Data, Distribution, Exclusion, and Ethics

We also acquired key demographic information including postal code, deprivation (based on the Index of Multiple Deprivation, which takes into account socioeconomic, occupational, housing, and environmental factors to estimate deprivation), age, gender, highest level of education, and occupation. The questionnaire, along with a detailed participant information sheet and consent form was distributed across the world *via* a secure weblink. We used several non-random sampling methods to reach respondents including: social media posting, emailing volunteer groups, and carrying out an online search of publicly available community group directories. The only exclusion criterion for the study was: people under 18 years of age. The questionnaire was ethically reviewed by the internal review committee in the Department of Landscape Architecture at the University of Sheffield (the authors’ academic institution).

### Statistical Analysis

To test the hypothesis that nature engagement i.e., duration and frequency of visits to nature, may positively influence a person’s attitudes toward microbes, we acquired a score from the word-association output by summing the positive, neutral and negative values given by each respondent—this was used as a proxy to indicate positive vs. negative attitude toward microbes. We then assigned the positive and negative scores into two groups and compared the mean duration and frequency of visits to nature of each group using the two-sample Mann–Whitney *U* test with continuity correction in R.

To test the hypothesis that nature connectedness influences people’s attitudes toward microbes, we built logistic regression models. For these models, an odds ratio (OR) of 1 or above equated to the predictor variable (nature connectedness score) increasing the odds of a positive attitude toward microbes. An OR < 1 equated to the predictor variable decreasing the odds of a positive attitude toward microbes. Answers from the question “do you consider microbes to be good” were coded into a “positive” category, and “do you consider microbes to be bad” were coded into a “negative” category, and these were then used in the regression models as binary dependent variables. We adjusted for several covariates including age, gender, deprivation, and level of education.

To test the hypothesis that basic knowledge of microbes influences people’s attitudes toward microbes, we assessed proportional differences between groups, in which respondents either did or did not identify different microbial groups (i.e., bacteria, viruses, fungi, algae, protozoa, and archaea) and their respective word-association scores (summing the negative, neutral and positive scores as a proxy to indicate a positive or negative attitude as a variable) using the 2-sample tests for equality of proportions with continuity correction in R. For example, three positive words = net positive score; two positive words and one negative or neutral = net positive score, and the reverse formula was used to acquire a net negative score.

## Results

**FIGURE 1:**
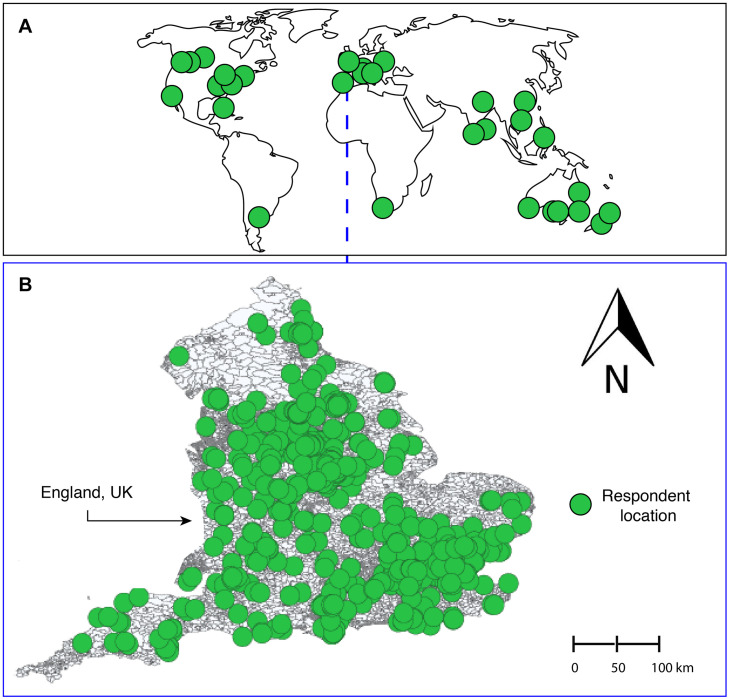
Distribution of respondents, whereby panel **(A)** shows the global distribution, and panel **(B)** shows England, United Kingdom—the geographical source of the majority of responses (*n* = 993).

A total of *n* = 1,184 respondents completed the questionnaire. A broad distribution of responses from across the world was acquired (Figure 1A); however, the main cluster (*n* = 993) was from England, United Kingdom (Figure 1B).

Respondents who identified as being female (*n* = 851 or 72%) outnumbered those who identified as being male (*n* = 331 or 28%), *trans* woman (*n* = 1 or 0.1%), and non-binary (*n* = 1 or 0.1%). There was also a skew toward respondents with a higher level of education (*n* = 847 or 72% with ≥ undergraduate degree). In terms of age, the distribution either side of the median was similar (*n* = 624 or 53% were ≥ 55 years old; and *n* = 560 or 47% were ≤ 54 years old).

### Nature Engagement, and Attitudes Toward Microbes

**FIGURE 2:**
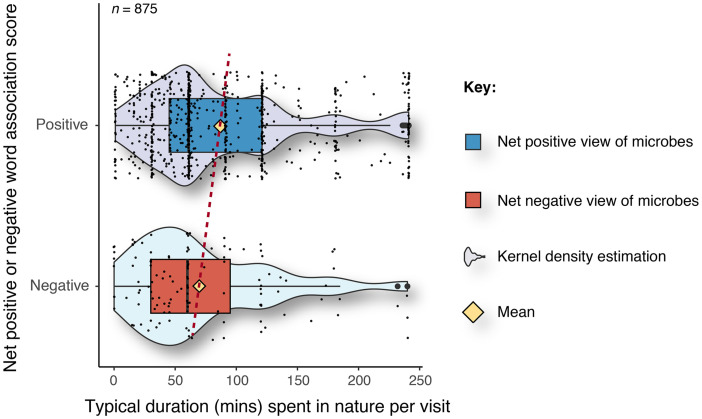
Typical duration spent in natural environments per visit for respondents with net positive and net negative word-association scores. The yellow diamond represents the mean value. The dashed red line is a visual aid to track the difference in means.

Our results show that respondents with a net positive word-association score for microbes (i.e., those who viewed microbes more positively) spent significantly more time per visit (x̊ = 87 min) to natural environments such as woodlands, parks, and meadows compared to respondents with a net negative word-association score for microbes (x̊ = 70 min) (W = 3,995, *p* ≤ 0.01) (Figure 2).

**FIGURE 3:**
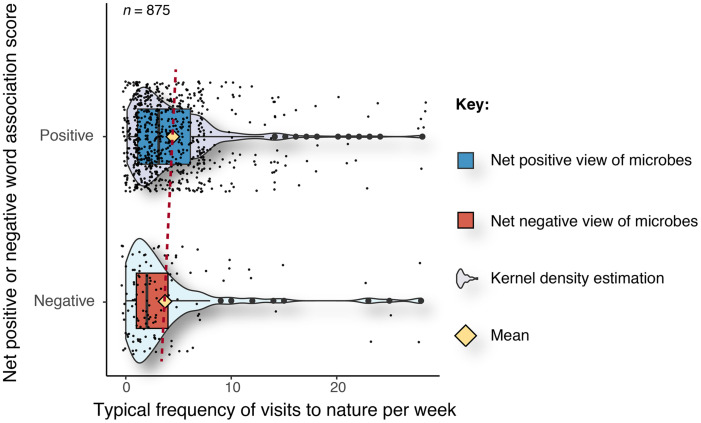
Typical frequency of visits to natural environments per week for respondents with net positive and net negative word-association scores. The yellow diamond represents the mean value. The dashed red line is a visual aid to track the difference in means.

Our results also show that respondents with a net positive word-association score for microbes visited natural environments such as woodlands, parks, and meadows significantly more often (x̊ = 4.2 visits in a given week) compared to respondents with a net negative word-association score for microbes (x̊ = 3.8 visits in a given week) (*W* = 3,935, *p* ≤ 0.01) (Figure 3).

### Nature Connectedness and Attitudes Toward Microbes

We found no association between nature connectedness (measured using the NR-6 Scale) and attitudes toward viruses (OR: 0.99 (0.95, 1.02) *p* = 0.54) or all other microbes (OR: 1.01 (0.89, 1.16) *p* = 0.86) (Table 1).

**TABLE 1:** Associations between attitudes toward microbes and nature connectedness, adjusting for relative deprivation, education, age and gender.

	**Model 1**	**Model 2**	**Model 3**	**Model 4**	**Model 5**
Viruses†					
Nature connectedness unadjusted¶	0.99 (0.95, 1.02) *p* = 0.54 N.S	–	–	–	–
Adjusted for IMD§	–	0.98 (0.89, 1.09) *p* = 0.70 N.S	–	–	–
Adjusted for education level	–	–	1.07 (0.96, 1.19) *p* = 0.21 N.S	–	–
Adjusted for age	–	–	–	0.97 (0.90, 1.05) *p* = 0.50 N.S	–
Adjusted for gender	–	–	–	–	1.13 (0.85, 1.52) *p* = 0.46 N.S
All other microbes†					
Nature connectedness unadjusted¶	1.01 (0.89, 1.16) *p* = 0.86 N.S	–	–	–	–
Adjusted for IMD§	–	0.98 (0.89, 1.09) *p* = 0.70 N.S	–	–	–
Adjusted for education level	–	–	1.19 (0.75, 1.88) *p* = 0.46 N.S	–	–
Adjusted for age	–	–	–	1.29 (0.94, 1.79) *p* = 0.12 N.S	–
Adjusted for gender	–	–	–	–	0.55 (0.17, 1.75) *p* = 0.60 N.S

*†Positive vs. negative view. Odds ratio and 95% CI reported. N.S, not significant. *n* = 1,184. §Adjusted by index of multiple deprivation (IMD) quintiles. ¶Based on nature relatedness-6 scale (NR-6).*

### Basic Microbial Literacy and Attitudes Toward Microbes

**FIGURE 4:**
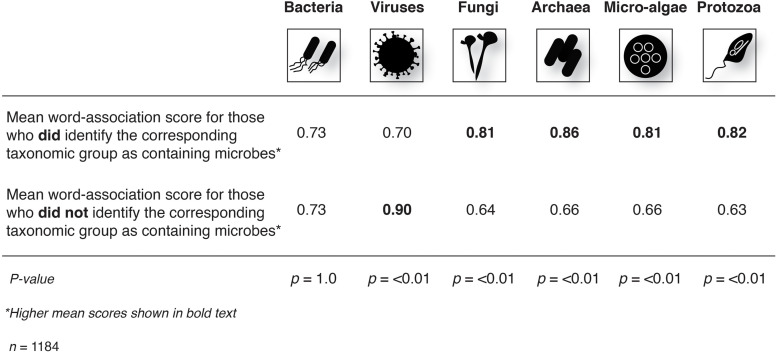
Differences in mean microbe word-associated scores for respondents who correctly identified a given taxa as being a microbe compared to those who did not identify the taxa as being a microbe. There were significantly higher (in positivity) word-association scores for respondents who correctly identified that fungi, archaea, micro-algae, and protozoa are microbes compared to those who did not.

Mean positive scores (derived from word-association) toward all microbes were significantly higher for those who correctly identified that fungi (*X*^2^ = 42.5, df = 1, *p* ≤ 0.01) archaea (*X*^2^ = 52, df = 1, *p* ≤ 0.01) micro-algae (*X*^2^ = 30, df = 1, *p* ≤ 0.01) and protozoa (*X*^2^ = 51, df = 1, *p* ≤ 0.01) were microbes compared to those who did not identify these groups as being microbes. Mean positive scores toward all microbes were significantly lower for those who identified that viruses were microbes compared to those who did not identify viruses as being microbes (*X*^2^ = 30.7, df = 1, *p* ≤ 0.01). There were no significant differences in scores between respondents who correctly identified bacteria as being microbes (*n* = 1124) compared to those who did not (*n* = 60) (*X*^2^ = < 0.01, df = 1, *p* = 1.0) (Figure 4).

## Discussion

Our study shows a significant positive relationship between nature engagement (a respondent’s duration and frequency in nature) and the respondents’ attitudes toward microbes. However, we found no association between nature connectedness (a person’s affective, cognitive and experiential connection with the natural world) (Cheung et al., 2020; Choe E. Y. et al., 2020) and attitudes toward microbes. Importantly, we found a significant relationship between knowledge of “lesser known” microbial groups (e.g., identifying that fungi, algae, protozoa, and archaea are microbes) and positive attitudes toward microbes. This study suggests that nature engagement and basic microbial literacy may be important in improving positive attitudes toward microbes. Further confirmatory research is required, with a focus on whether these potential changes to attitudes translate to changes in germaphobia-associated behaviors.

As mentioned, nature engagement significantly associated with positive attitudes toward microbes. This finding supports our first hypothesis, and is corroborated by other (non-microbiological) work that suggests nature engagement may reduce fears of “creepy crawlies” and help foster respectful and positive attitudes toward nature (Hosaka et al., 2017; Chawla, 2020). It is important to note that the directionality of the relationship is unknown (i.e., whether spending more time in nature helps to establish more positive attitudes toward microbes, or whether other factors related to more positive attitudes increase the likelihood of spending more time in nature). Conceivably, being less averse to microbes could increase one’s desire to spend time in environments with natural features such as plants and soil—key sources of dense microbial communities (Liddicoat et al., 2019; Robinson et al., 2020). On the other hand, a greater habituation to these kinds of environments and an affinity for natural environments with its diverse life-forms could conceivably reduce one’s aversion to microbes in general (as shown with “macro” organisms). It is important to acknowledge here that spending time in natural environments exposes us to a diverse suite of microbial communities (Robinson et al., 2020; Selway et al., 2020) that are thought to have important beneficial effects on our health (Haahtela, 2019; Renz and Skevaki, 2020). Therefore, whatever the actual directionality of the proposed relationship is (which requires further research to determine), it is likely to have an important impact on our health and could help to ameliorate the negative consequences of germaphobia (e.g., immune dysfunction) (Rook et al., 2003). In one direction (i.e., contingent on factors related to more positive attitudes toward microbes increasing the likelihood that we will spend more time in nature), we could potentially gain the many benefits associated with nature engagement. These include improvements in immune health (Li, 2010; Rook, 2013), mental health (Birch et al., 2020; Callaghan et al., 2020), and cardiovascular health (Yeager et al., 2020). In the alternative direction (i.e., spending more time in natural environments which may help to establish more positive attitudes toward microbes), we can hypothesize that our positive attitudes toward microbes could conceivably reduce the likelihood that we carry out mass (non-targeted) sterilization of our local environments, which could also have important implications for our health (Jun et al., 2018; Parks et al., 2020; Prescott, 2020; Renz and Skevaki, 2020). This hypothesis requires further research and would benefit from the collection of data on people’s actions (e.g., related to environmental avoidance and sterilization). This relationship could also be non-dichotomous (or potentially even a virtuous loop) in the sense that our positive attitudes toward microbes may predispose us to spend more time in nature—an act that may enhance our positive attitudes toward microbes, and the feedback continues. This theoretical relationship warrants further research.

Given that we have shown that nature engagement (duration and frequency in nature) associates with positive attitudes toward microbes, it would perhaps be expected that nature connectedness may also associate with positive attitudes toward microbes (our second hypothesis). Studies have shown that people who exhibit higher levels of nature connectedness are more likely to spend time in and engage with natural environments (Capaldi et al., 2014, 2015), and reciprocally, spending time in nature can enhance one’s nature connectedness (Nisbet et al., 2019; Chawla, 2020). However, the results of our study show that no significant relationship existed between the nature connectedness of our respondents and their attitudes toward microbes. This could be confounded by other factors, however, age, gender, education and deprivation were controlled for with similar non-significant results. It may simply be that a person’s affective, cognitive and experiential connection with nature is not an important factor in predicting one’s attitude toward microbes. We can only speculate and say that the invisibility of microbes to the human eye could conceivably negate the affective, cognitive and experiential connection that one may establish with, for example, charismatic fauna or aesthetically-appealing flora. There is a deficit in research on people’s emotional and cognitive connection with the invisible constituents of the natural world, and as such, future studies focusing on this relationship would be valuable. It is worthwhile to point out that in contrast to macro-level organisms (e.g., birds and trees), it is only recently—evolutionarily speaking—that humans have been aware of diverse microscopic lifeforms, and only in the past few decades have we been able to comprehensively characterize microbial communities and understand their ecology (Hugenholtz and Tyson, 2008). At this stage, it can only be speculated that this may have an effect on the relationship between nature connectedness and attitudes toward microbes, that is, *via* a lack of a developed emotional link through sense (e.g., sight, sound, touch)-stimuli interactions over evolutionary timescales. Alternatively, this result could be a facet of the nature connectedness instrument used (the NR-6 Scale). Perhaps a more detailed version of the instrument such as the 17-item Connectedness to Nature Scale (CNS) (Mayer and Frantz, 2004) would reveal alternative findings. This warrants further research.

Finally, our study shows a significant relationship between basic level of microbial literacy and attitudes toward microbes, which supports our third hypothesis. Previous work has suggested that environmental knowledge can positively affect attitudes toward nature (Sat Gungor et al., 2018; Choe J. H. et al., 2020), although other research suggests this is not important (Qomariah and Prabawani, 2020). In our study, respondents who correctly identified that lesser publicized (as microbes) organisms – such as algae, fungi, archaea, and protozoa – were microbes, showed higher positivity scores toward microbes. This implies that basic microbial literacy may be an important factor in the formation of a person’s attitudes toward microbes, and thus could potentially influence the onset of germaphobia. Determining whether any potential influences on people’s attitudes subsequently translates into “germaphilic” or microbe-appreciative behaviors, requires further research. Interestingly, mean positive scores toward all microbes were significantly lower for those respondents who identified that viruses were microbes compared to those who did not identify viruses as being microbes. Although further research is needed, one explanation could be that the COVID-19 (virus) pandemic had an effect on people’s overall view of microbes. This may be unsurprising given the damage the pandemic has caused and the multi-pronged approach taken to try and eliminate the SARS-CoV-2 virus. However, it could conceivably have negative cascading effects on our health by contributing to broader germaphobia.

Microbes are the foundations of our ecosystems and are essential to the survival of all life on Earth (Cavicchioli et al., 2019). We now have the technology to easily characterize and learn about these diverse invisible communities that continuously surround us, providing essential ecosystem services. Although further research is required to build upon our preliminary findings, it is conceivable that in the future, strategies that aim to enhance positive attitudes toward microbes could include the promotion of nature engagement (spending more time and more often in nature), which has several important co-benefits for health and wellbeing (Rook, 2013; Birch et al., 2020). Moreover, perhaps in an educational context, greater emphasis can be placed on microbial literacy moving into the future. With a more nuanced awareness of, and mutually-advantageous relationship with these diverse, underappreciated, and indispensable lifeforms, germaphobia-associated attitudes can potentially be reduced, while still maintaining the critically important targeted-hygiene and efforts to control infectious diseases.

### Limitations

Our study has some important limitations. Firstly, the results in the study are correlational. Therefore, strict inferences of causation are not possible. Along similar lines, inferences regarding the directionality of the relationships are also not possible. Non-random sampling methods were used in this study. This means accurate calculations of error and representativeness are not possible. Perhaps one of the most important limitations is that self-reported data collection methods come with inherent biases. For example, responder bias—where participants, either intentionally or by accident, choose an untruthful or inaccurate answer, or where people who consider nature important are over-represented in the study. We acknowledge our attitude assessment was limited, and future studies would benefit from investigating behaviors such as environmental avoidance and sterilization. Further controlled research is required to fully unravel the complexities of the observed relationships. There was also a deficit of samples from outside of England, United Kingdom. The study would have benefited from the inclusion of additional international georeferenced samples to be representative on a wider scale. Temporally-objective nature-engagement data that represents scenarios before the COVID-19 pandemic, during the pandemic, and after the pandemic would also bring considerable value.

## Conclusion

This study suggests that basic microbial literacy and nature exposure may be important in reducing/preventing germaphobia-associated attitudes. As the rise of immune-related disorders and mental health conditions have been linked to germaphobia, reduced biodiversity, and non-targeted sterilization, our findings point to a simple strategy to potentially help ameliorate these negative consequences, although further research is required to explore this in greater detail. Indeed, a greater emphasis on microbial literacy and promoting time spent in nature could potentially be useful in promoting resilience in human health and more positive/constructive attitudes toward the foundations of our ecosystems—the microorganisms.

## Data Availability Statement

The datasets presented in this study can be found in online repositories. The names of the repository/repositories and accession number(s) can be found below: United Kingdom Data Service ReShare: https://reshare.ukdataservice.ac.uk/ Data Collection: 854604.

## Ethics Statement

The studies involving human participants were reviewed and approved by University of Sheffield; Department of Landscape Architecture Review Committee. The patients/participants provided their written informed consent to participate in this study.

## Author Contributions

JR, RC, and AJ contributed to the conception and design of the study and manuscript internal critical review process and revisions. JR conducted the data analysis, wrote the manuscript, produced the figures, and data visualizations. All authors read and approved the submitted version.

## Conflict of Interest

The authors declare that the research was conducted in the absence of any commercial or financial relationships that could be construed as a potential conflict of interest.

## Supplementary Material

Supplementary Appendix A Online survey questions.
