# Supplementary material for: Germaphobia! Does Our Relationship With and Knowledge of Biodiversity Affect Our Attitudes Toward Microbes?
Source: Front Psychol. 2021 Jun 30;12:678752. doi: 10.3389/fpsyg.2021.678752 (PMC8278522; doi:10.3389/fpsyg.2021.678752)
Supplement: Supplementary Appendix A — Online survey questions. [file Data_Sheet_1.pdf]

Supplementary Materials

Online survey questions

- What is your age?
  - o Dropdown menu of ages groups (18-24; 25-34; 35-44; 45-54; 55-64; 65-74; 85+)
- What is your gender?
  - o Dropdown menu with additional textbox to include other genders
- What is/was your main occupation?
  - o Open textbox
- What is your level of education?
- Dropdown menu of education levels (from no qualifications to PhD)
- What country do you live in?
  - o Open textbox
- What is your postal/zip code?
  - o Open textbox
- How many times would you visit any natural environments (e.g., parks, woodlands, the beach) in a typical week before the COVID-19 pandemic?
  - o Slider bar from 0 to 100 (times)
- Approximately how long would you spend in any natural environment per visit before the COVID-19 pandemic?
  - o Slider bar from 0 to 1000 (minutes)
- Select all of the organisms that you consider to be microbes (micro-organisms):
  - o Multiple selection tick boxes including the following options: Bacteria, Viruses, Archaea, Protozoa, Fungi, Algae
- Do you consider viruses to be:
  - o Single selection (good; bad; some are, good some are bad; neither good or bad)
- Do you consider all other microbes (micro-organisms) to be:
  - o Single selection (good; bad; some are, good some are bad; neither good or bad)
- From the list below, choose 3 words that you think best describe microbes:

1. Useful	2. Disease	3. Neutral
4. Death	5. Beneficial	6. Beautiful
7. Healthy	8. Living	9. Nuisance
10. Mobile	11. Pandemic	12. Essential
13. Toxic	14. Nature	15. Life

- Options 1, 5, 6, 7, and 12 above were coded into a Positive category; Options 2,4,9,11, and 13 were coded into a Negative category; Options 3,8,10,14, and 15 were coded into a Neutral category.

- How much do you agree or disagree with the following (i.e. using the Nature Relatedness 6 Scale):
  Select one for each line (options: Disagree, Slightly Disagree, Neutral, Slightly Agree, Agree)

- I feel very connected to all living things and the earth
- I always think about how my actions affect the environment
- My relationship to nature is an important part of who I am
- My connection to nature and the environment is a part of my spirituality
- My ideal holiday/vacation spot would be a remote, wilderness area
- I take notice of wildlife wherever I am
